# Symptom network analysis in breast cancer patients: A scoping review

**DOI:** 10.1371/journal.pone.0336793

**Published:** 2025-11-24

**Authors:** Wenxi Li, Xiaoyan Wang, Qin Yu, Wen Zhou, Qianmei Zhong

**Affiliations:** 1 Department of Oncology, Mianyang Central Hospital, Affiliated with the School of Medicine, University of Electronic Science and Technology of China, Mianyang, China; 2 State Key Laboratory of Ultrasound Medical Engineering, School of Biomedical Engineering, Chongqing Medical University, Chongqing, China; 3 Department of Immunology, Mianyang Central Hospital, Affiliated with the School of Medicine, University of Electronic Science and Technology of China, Mianyang, China; All India Institute of Medical Sciences, INDIA

## Abstract

Existing research on symptom management in patients with breast cancer has predominantly focused on symptom cluster construction. However, the mechanisms underlying symptom interactions remain unclear. This disparity has hindered the development of efficient and precise strategies for symptom management. Symptom network analysis offers a novel approach by visualizing the complex interrelationships and interaction strengths among symptoms, thereby enabling the identification of central and bridge symptoms. This scoping review aimed to map the symptom network structure in patients with breast cancer and identify the core and bridge symptoms, thereby providing a theoretical foundation for developing personalized and precise symptom management strategies in clinical practice. Searches of PubMed, Web of Science, Cochrane Library, Embase, Elton B. Stephens Company (EBSCO), China National Knowledge Infrastructure (CNKI), Wanfang Data, and SinoMed were conducted to retrieve relevant literature on breast cancer symptom network analysis published from the time of database initiation to January 31, 2025, and assess the data. Thirteen papers were included, of which five were in Chinese and eight were in English. Symptom-related assessment tools included 13 types of single-symptom assessment scales and multi-symptom assessment scales, of which five studies used two or more scales; a total of 10 core and bridge symptoms were extracted, of which fatigue was the crucial core and bridge symptom. The symptom networks of breast cancer patients differ and change dynamically under varied treatment modalities. Although research on the symptom networks of patients with breast cancer has progressed, pitfalls such as unbalanced development and non-uniform research methods remain. Large-scale longitudinal studies are required to frame dynamic symptom networks, develop specific measurement tools, and combine latent variable analyses to construct heterogeneous symptom networks that will facilitate precise symptom management.

## Introduction

According to global cancer statistics, breast cancer is the second most common malignancy worldwide and the leading cause of cancer-related mortality among women, posing a significant threat to their health [1]. The current treatment options for breast cancer primarily include surgery, radiotherapy, chemotherapy, immunotherapy, molecular-targeted therapy, and endocrine therapy [2]. Due to the disease itself and the side effects of treatment, patients with breast cancer frequently experience multiple concurrent symptoms, such as fatigue, pain, loss of appetite, anxiety, and sleep disturbances [3–4]. Compared with individual symptoms, the concurrence of multiple symptoms can exert a profound negative impact on patients’ quality of life and functional status, potentially leading to treatment delays or discontinuation, increased healthcare costs, and an overall decline in health outcomes [5–6]. Existing research on symptom management in patients with breast cancer has predominantly focused on symptom cluster construction [7–8], with limited attention given to the mechanisms underlying symptom interactions. This inconsistency has hindered the development of efficient and precise strategies for symptom management. Symptom network analysis offers a novel approach by visualizing complex interrelationships and interaction strengths among symptoms, thereby enabling the identification of central and bridge symptoms [9]. Core symptoms are defined as those exhibiting the strongest connectivity within the symptom network and playing a pivotal role in triggering the co-occurrence of multiple symptoms. Bridge symptoms, in contrast, serve as critical connectors between distinct symptom clusters and represent potential therapeutic targets for disrupting maladaptive interactions across networks. Targeted interventions directed at core and bridge symptoms may effectively attenuate cascading effects among interrelated symptoms throughout the network structure, thereby offering a novel framework for advancing the science of symptom management [10].

This scoping review aimed to map the symptom network structure in patients with breast cancer and identify the core and bridge symptoms, thereby providing a theoretical foundation for developing personalized and precise symptom management strategies in clinical practice.

## Materials and methods

This scoping review adhered to the Preferred Reporting Items for Systematic reviews and Meta-analyses–PRISMA checklist. This research is exempt from the Research Ethics Board (REB).

### Identification of relevant literature

#### Eligibility criteria.

Based on the PCC framework (Participants, Concept, Context) [11], the inclusion criteria for this review were as follows: (1) Participants: studies involving patients with breast cancer, with no restrictions on age, gender, or ethnicity; (2) Concept: studies focused on symptom network analysis, including symptomics; and (3) Context: no limitations regarding treatment stage or healthcare setting. The exclusion criteria were as follows: (1) duplicate publications; (2) studies for which the full text was unavailable; and (3) studies published apart from Chinese or English.

#### Information sources & search tools.

A comprehensive search strategy combining Medical Subject Heading terms and free-text keywords was employed to retrieve relevant literature from the following databases: PubMed, Web of Science, Cochrane Library, Embase, EBSCO, CNKI, Wanfang Data, and the Chinese Biomedical Literature Database (CBM). Studies published from database inception till January 31, 2025, focusing on symptom network analysis in patients with breast cancer were included. The English search terms included were: “breast neoplasm/breast cancer/ breast tumor*/breast carcinoma*/mammary cancer*,” “symptom/symptomatology/symptomology,” “network analysis/network structure”. Taking PubMed as an example: (((“Breast Neoplasms”[Mesh]) OR (breast cancer[Title/Abstract] OR breast tumor*[Title/Abstract] OR breast carcinoma*[Title/Abstract] OR mammary cancer*[Title/Abstract])) AND (symptom[Title/Abstract] OR symptomatology[Title/Abstract] OR symptomology[Title/Abstract])) AND (network analysis[Title/Abstract] OR network structure[Title/Abstract]).

#### Study selection and data extraction.

Duplicate records were removed using EndNote 21. Two reviewers (Wenxi Li and Xiaoyan Wang) independently conducted an initial screening of titles and abstracts, followed by a full-text review of the studies that met the inclusion criteria. Any discrepancies during the selection process were resolved through discussions with a third reviewer (Qianmei Zhong). A data extraction sheet was developed using Microsoft Excel. Two reviewers independently extracted the following information from the included studies: first author, year of publication, country or region, study design, sample size, participant characteristics, symptom assessment tools, as well as identified central and bridge symptoms.

## Results

### Literature search results

**Fig 1:**
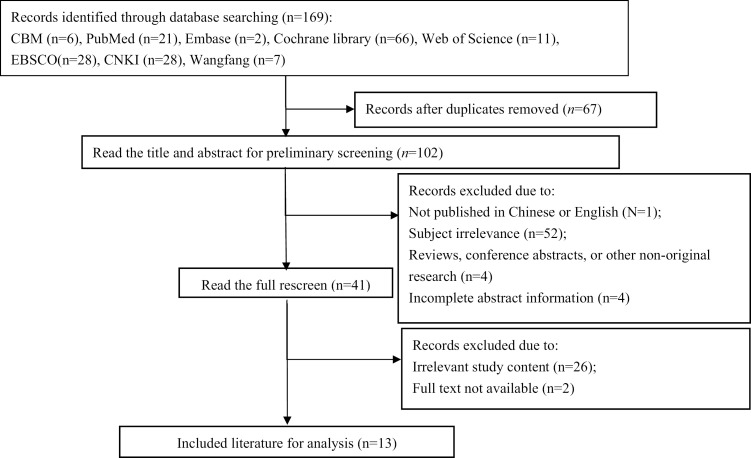
Flow chart for literature search and screening.

A total of 169 records were initially identified from the databases. After removing the duplicates, 102 articles remained. After title and abstract screening, 41 articles were retained for full-text review. Ultimately, 13 studies met the inclusion criteria and were included in the final analysis [12–24]. The study selection process is illustrated in Fig 1.

### Basic characteristics of the included studies

The included studies were published between 2023 and 2025 and conducted in China (n = 12) and South Korea (n = 1). All studies were quantitative in nature, with 12 cross-sectional studies [12–15,17–24] and one longitudinal study [16]. The treatment modalities included chemotherapy, radiotherapy, endocrine therapy, and combined therapies. A total of 5,633 adult female participants (aged ≥18 years) were included in the study. The detailed characteristics are presented in Table 1.

**Table 1:** Basic Characteristics of the Included Studies (n = 13).

Included literatures	Year	Country	Study design	Population description	Sample size	Symptom assessment tools
Zhang et al. [12]	2024	China	Cross-sectional	Postoperative breast cancer patients receiving chemotherapy	327	MDASI, FACT-B
He et al. [13]	2024	China	Cross-sectional	Breast cancer patients receiving endocrine therapy	330	C-BCPT
Zha et al. [14]	2024	China	Cross-sectional	Postoperative breast cancer patients receiving radiotherapy	168	MSAS-Ch
Lei et al. [15]	2024	China	Cross-sectional	Home-based breast cancer patients after chemotherapy discharge	478	MDASI-C
He et al. [16]	2024	China	Longitudinal	Breast cancer patients receiving chemotherapy	467	FACT-B, Breast Cancer Chemotherapy Symptom Scale
Cai et al. [17]	2023	China	Cross-sectional	Breast cancer patients (< 60 years old) receiving chemotherapy	1033	PROMIS-57, PROMIS-Cognitive Function Short Form
Jing et al. [18]	2023	China	Cross-sectional	Breast cancer patients receiving endocrine therapy	613	FACT-ES
Liang et al. [19]	2024	China	Cross-sectional	Breast cancer patients receiving chemotherapy	468	MDASI
Chang et al. [20]	2024	China	Cross-sectional	Breast cancer patients receiving chemotherapy	292	NRS, PSQI, CFS, HADS
He et al. [21]	2025	China	Cross-sectional	Breast cancer patients receiving endocrine therapy	406	C-BCPT
Kim et al. [22]	2024	Korea	Cross-sectional	Breast cancer patients with treatment-induced menopausal symptoms	250	EORTC QLQ-C30, EORTC QLQ-BR45
Xiao et al. [23]	2025	China	Cross-sectional	Elderly breast cancer patients (≥ 65 years old)	481	EORTC QLQ-C30
Teng et al. [24]	2024	China	Cross-sectional	Breast cancer patients receiving chemotherapy	320	MDASI-C

MDASI: M. D. Anderson Symptom Inventory; MDASI-C: Chinese version of the M. D. Anderson Symptom Inventory; FACT-B: Functional Assessment of Cancer Therapy–Breast; C-BCPT: Chinese version of Breast Cancer Prevention Trial Symptom Checklist; MSAS-Ch: Chinese version of Memorial Symptom Assessment Scale; PROMIS: Patient-Reported Outcomes Measurement Information System; PROMIS-CF: PROMIS Cognitive Function Short Form; FACT-ES: Functional Assessment of Cancer Therapy–Endocrine Subscale; NRS: Numeric Rating Scale; PSQI: Pittsburgh Sleep Quality Index; CFS: Cancer Fatigue Scale (Chinese version); HADS: Hospital Anxiety and Depression Scale; EORTC QLQ-C30: European Organization for Research and Treatment of Cancer Quality of Life Questionnaire–Core 30; EORTC QLQ-BR45: EORTC QLQ Breast Cancer Module.

### Symptom assessment tools used in the included studies

Thirteen symptom assessment tools were used across the 13 included studies, comprising four single-symptom scales and nine multi-symptom instruments. The most commonly used multi-symptom tools were the M.D. Anderson Symptom Inventory (MDASI, n = 2), Chinese version of the MDASI (MDASI-C, n = 2), Functional Assessment of Cancer Therapy–Breast (FACT-B, n = 2), Chinese version of the Breast Cancer Prevention Trial Symptom Scale (C-BCPT, n = 2), and European Organization for Research and Treatment of Cancer Quality of Life Questionnaire–Core 30 (EORTC QLQ-C30, n = 2). Four studies [12,16,17,20] used two or more instruments in combination; however, each used a unique combination of scales.

### Characteristics of symptom networks in included studies

The symptom networks constructed in the included studies encompassed physiological, psychological, and social dimensions. All studies utilized R software for network construction. The key node metrics analyzed included strength centrality, closeness centrality, betweenness centrality, and expected influence. As there is currently no universally accepted standard for determining the minimum sample size in symptom network models, the sample sizes reported in the literature included in this article exhibited considerable variability. Notably, the study conducted by Zha et al [14]. had the smallest sample size, which was determined based on a criterion of “5-6 individuals corresponding to each symptom node,” ultimately including 168 patients. The findings indicate that the symptom network constructed from this sample size demonstrated good stability and accuracy. Among the included studies, 12 constructed cross-sectional symptom networks [12–15,17–24], whereas one developed a longitudinal (dynamic) symptom network [16].

### Core and bridge symptoms identified in included studies

A total of 10 core symptoms were identified across the studies included, with fatigue being the most frequently reported (n = 7) [14–17,19,22,23], followed by difficulty in concentrating (n = 2) [13,21]. Ten bridge symptoms were also extracted, primarily fatigue (n = 5) [12,13,15,16,22] and poor appetite/anorexia/decreased appetite (n = 3) [12,15,19]. Four studies [17,20,21,24] conducted subgroup analyses and identified core symptoms within each subgroup. The core symptoms in the subgroups mainly included pain (n = 3) [17,20,21] and fatigue (n = 2) [17,21]. Among the studies included in this review, there were variations in the methods used to identify core symptoms and bridge symptoms. For core symptom identification, different studies employed distinct centrality indices. Four studies [13,14,18,21] solely relied on strength as the criterion for identifying core symptoms, whereas three [16,19,24] adopted a composite approach using strength, closeness, and betweenness. In the identification of bridge symptoms, four studies [14–16,22] used betweenness centrality as the primary indicator, whereas three used indicators such as the Expected Influence, Coefficient of Bridges, Bridge Strength, and Bridge Compactness for identification [12,13,19]. The details are presented in Table 2.

**Table 2:** Core symptoms, bridging symptoms and research methods covered in the literature (n = 13).

Included literatures	Core symptoms	Subgroup core symptoms	Research Methods for Core Symptoms	Bridge symptoms	Subgroup bridge symptoms	Research methods for bridge symptoms
Zhang et al. [12]	—	—	—	Fatigue, poor appetite, distress	—	Coefficient of Bridges, Bridge Strength
He et al. [13]	Difficulty concentrating	—	Strength	Fatigue, weight gains, lack of interest in sexual activity	—	Bridge Dtrength
Zha et al. [14]	Fatigue	—	Strength	Skin changes	—	Betweenness
Lei et al. [15]	Fatigue, Sadness, Nausea	—	Closeness	fatigue, anguish, anorexia	—	Betweenness
He et al. [16]	Sleep difficulty (pre-chemo), Appetite loss & pain (post 1^st^ chemo), fatigue (post 3^rd^ & 6^th^ chemo)	—	Strength, Closeness, Betweenness	Depression (pre-chemo), headache (post 1^st^ chemo), fatigue (post 3^rd^ & 6^th^ chemo)	—	Betweenness
Cai et al. [17]	Fatigue	Helplessness and fatigue (severe); pain and despair (moderate anxiety-depression-pain); pain and fatigue (mild)	Strength, Closeness	—	—	—
Jing et al. [18]	Mood swings, irritability	—	Strength	—	—	—
Liang et al. [19]	Fatigue	—	Strength, Closeness, Betweenness	Sleep disturbance, appetite loss	—	Expected Influence, Coefficient of Bridges, Bridge Strength, Bridge Compactness
Chang et al. [20]	Panic	Tension and pain (high burden); Panic (low burden)	Strength, Closeness	—	—	—
He et al. [21]	Difficulty in concentrating	Fatigue (low distress); Pain (high distress)	Strength	—	—	—
Kim et al. [22]	Fatigue	—	Strength, Closeness	Fatigue	—	Betweenness
Xiao et al. [23]	Fatigue	—	Expected impact coefficient	—	—	—
Teng et al. [24]	—	Appetite loss (low self-advocacy); distress (moderate & high self-advocacy)	Strength、Closeness, Betweenness	—	—	—

## Discussion

### Symptom networks in breast cancer patients are complex, fatigue may serve as a key target for intervention

Symptoms such as fatigue, sleep disturbance, decreased appetite, nausea, and anguish are highly prevalent and often severe among patients with breast cancer [12–24], suggesting a widespread burden of symptom distress that warrants comprehensive assessment and management. Although the symptoms experienced by breast cancer patients may differ depending on the treatment methods employed, numerous studies have consistently identified that fatigue ranks highest in terms of incidence or severity when utilizing various symptom assessment tools [12–15,22,23]. Fatigue has also been frequently identified as both a core symptom [14–17,19,21–23] and a bridge symptom [12,13,15,16,22] within symptom networks.

Given its centrality and connectivity in the symptom network, fatigue should be prioritized as a key intervention target in the symptom management of patients with breast cancer. Non-pharmacological interventions are currently recommended as first-line strategies for fatigue and include exercise therapy, psychological support, cognitive behavioral therapy, and nutritional interventions [25].

### Symptom networks in breast cancer patients are dynamic: The need to strengthen research on longitudinal networks

In a longitudinal study by He et al. [16] it was demonstrated that the symptom network structure in patients with breast cancer undergoing chemotherapy changes dynamically over time, with variations in both core and bridge symptoms at different treatment stages. Prior to chemotherapy, the median number of reported symptoms was four, with sleep difficulty as the core symptom and depressed mood as the bridge symptom. After the first chemotherapy cycle, the median number of symptoms increased to 11, with appetite loss and pain emerging as core symptoms and headache as a bridge symptom. By the third and sixth chemotherapy cycles, the median symptom count further increased to 12 and 13, respectively, with fatigue consistently identified as both a core and bridge symptom. These findings suggest that symptom networks evolve throughout the treatment trajectory and highlight the importance of stage-specific dynamic symptom management strategies.

However, most studies included in this review were cross-sectional in nature [12–15,17–24] and constructed symptom networks based on data collected at a single time point, referred to as cross-sectional or contemporaneous symptom networks [26]. While useful, these networks cannot capture symptom trajectories or infer the causality between symptoms. In contrast, dynamic symptom networks built from repeated measures across multiple time points in the same cohort can reflect temporal shifts in symptom associations and reveal their underlying mechanisms [27].

In summary, future research should prioritize longitudinal designs to construct dynamic symptom networks. This approach enables a better understanding of symptom evolution and interrelationships over time, ultimately providing personalized and temporally adaptive symptom management in breast cancer care.

### Lack of specificity in symptom assessment tools among breast cancer patients: A need for standardization

The symptom assessment tools used in symptom network studies of breast cancer patients vary widely, encompassing both single-symptom scales and multi-symptom instruments. In most cases, researchers independently select instruments based on study objectives, resulting in inconsistencies in applicability and standardization. The current tools are primarily designed for general symptom assessment in patients with breast cancer.

For instance, in studies involving patients undergoing endocrine therapy, He et al. [13] used the C-BCPT and identified “difficulty concentrating” as the core symptom, while Jing et al. [18], using the FACT-ES, found “emotional lability” and “irritability” to be core symptoms. Liang et al. [19] applied the MDASI, identifying “fatigue” as the core symptom, whereas Chang et al. [20], using four separate single-symptom tools (NRS, PSQI, CFS, HADS), identified “panic” as the core symptom. These discrepancies indicate that variations in assessment tools may result in significant differences in the structure and interpretation of symptom networks, even among similar patient populations. Such inconsistencies are not conducive to clinical practice, particularly when formulating effective symptom management strategies based on these assessments.

Therefore, the development of breast cancer–specific, sensitive, and standardized symptom assessment instruments is urgently required. A unified approach to tool selection and application will enhance the comparability and accuracy of symptom network analyses, and ultimately improve symptom management strategies in both research and clinical settings.

### Combining latent variable analysis with network analysis enhances understanding of symptom network heterogeneity across subgroups

Given the substantial individual variability in symptom expression among patients with breast cancer, identifying heterogeneous subgroups based on symptom profiles may be the key to optimizing symptom management strategies [28]. Latent variable analysis enables the identification of unobserved subpopulations within a larger cohort that share similar observable characteristics, but differ in their underlying symptom structures [29], offering valuable insights into symptom heterogeneity.

Several studies included in this review employed integrated approaches that combine latent variable modeling, specifically latent class analysis (LCA) or latent profile analysis (LPA), with network analysis. In these studies, LCA or LPA was used to classify patients into symptom-based subgroups, followed by the construction of separate symptom networks for each subgroup. For example, Cai et al. [17] utilized LCA in combination with network analysis and found that core symptoms varied significantly across subgroups of patients undergoing chemotherapy. Similarly, He et al. [21] and Teng et al. [24] applied LPA and observed differences in core symptoms between subgroups. It is evident that the symptom networks of subgroups exhibit greater heterogeneity. Future research should aim to enhance both the depth and breadth of latent variable analysis combined with network analysis in studying symptom networks among breast cancer patients. This integrated analytical approach can be employed to more accurately delineate the classification criteria for symptom subgroups in breast cancer patients with varying clinical characteristics, thereby providing methodological support for the refined identification of these subgroups. Furthermore, the subgroup-specific characteristics of symptom networks revealed through this method can offer a scientific foundation for developing targeted intervention strategies.

Specifically, differentiated intervention strategies should be formulated based on the unique symptom profiles of various subgroups. The core symptoms identified within each subgroup ought to serve as primary targets for intervention, prioritizing those key symptoms that exert significant influence on patients’ overall symptom burden and are likely to trigger the transmission and exacerbation of other symptoms. Concurrently, by leveraging the connection characteristics associated with bridge symptoms within these subgroups, it is essential to regulate the strength of associations between bridge symptoms and other related symptoms to disrupt cyclical interactions among them, ultimately leading to a reduction in the overall symptom burden experienced by patients within these subgroups.

### Pain should be prioritized as the primary intervention target for patients experiencing a significant symptom burden in breast cancer

Chang et al [20] and He et al [21] demonstrated that, following the sub-group classification of symptom burden in breast cancer patients, pain emerges as the core symptom within the high symptom burden group. This finding indicates that pain in breast cancer patients does not occur in isolation; rather, it is significantly intertwined with other distressing symptoms such as fatigue, sleep disturbances, and emotional abnormalities. Pain serves as a “core node” linking these symptoms and acts as a critical marker for distinguishing between high distress groups and low/moderate distress groups. Consequently, if pain is not effectively managed, alleviating other symptoms will be considerably hindered, making it challenging to reduce the overall symptom burden experienced by patients. This underscores the importance of targeting pain management as a key intervention strategy for improving outcomes among breast cancer patients with high symptom distress. A comprehensive and accurate assessment represents both an essential initial step and a fundamental guarantee for effective cancer pain management [30]. Medical professionals should perform thorough evaluations regarding the type, severity, and duration of patients’ pain experiences. In addition to standardized pharmacological treatments, non-pharmacological interventions—such as music therapy and mindfulness practices—should also be integrated into care strategies [31]. Furthermore, traditional Chinese medicine approaches like acupuncture and moxibustion hold significant application value in this context. The study conducted by Li et al. [32] validated the efficacy of these methods in managing pain among liver cancer patients; thus, providing valuable insights for implementing similar interventions aimed at addressing pain in breast cancer populations.

## Conclusions

The symptom network in patients with breast cancer is complex and evolves dynamically across different stages of treatment. Core and bridge symptoms play pivotal roles in the network and represent promising targets for alleviating the overall symptom burden. Although current research on symptom network analysis in this population has yielded important insights, further longitudinal studies are required to construct dynamic symptom networks. In addition, the development of disease-specific assessment tools and integration of latent variable analysis into network modeling are crucial for advancing precise and effective symptom management strategies.

## Supporting information

S1 Fig Flow chart for literature search and screening. (DOCX)

S1 Table Basic characteristics of the included studies (n = 13). (DOCX)

S2 Table Core symptoms, bridging symptoms and research methods covered in the literature (n = 13). (DOCX)

S1 File PLOS one human subjects research checklist. (DOCX)

S2 File PRISMA-ScR-fillable-checklist. (DOCX)
