# Supplementary figures and images for: Symptom network analysis in breast cancer patients: A scoping review
Source: PLoS One. 2025 Nov 24;20(11):e0336793. doi: 10.1371/journal.pone.0336793 (PMC12643262; doi:10.1371/journal.pone.0336793)

S1 Fig 1. Flow Chart for Literature Search and Screening.


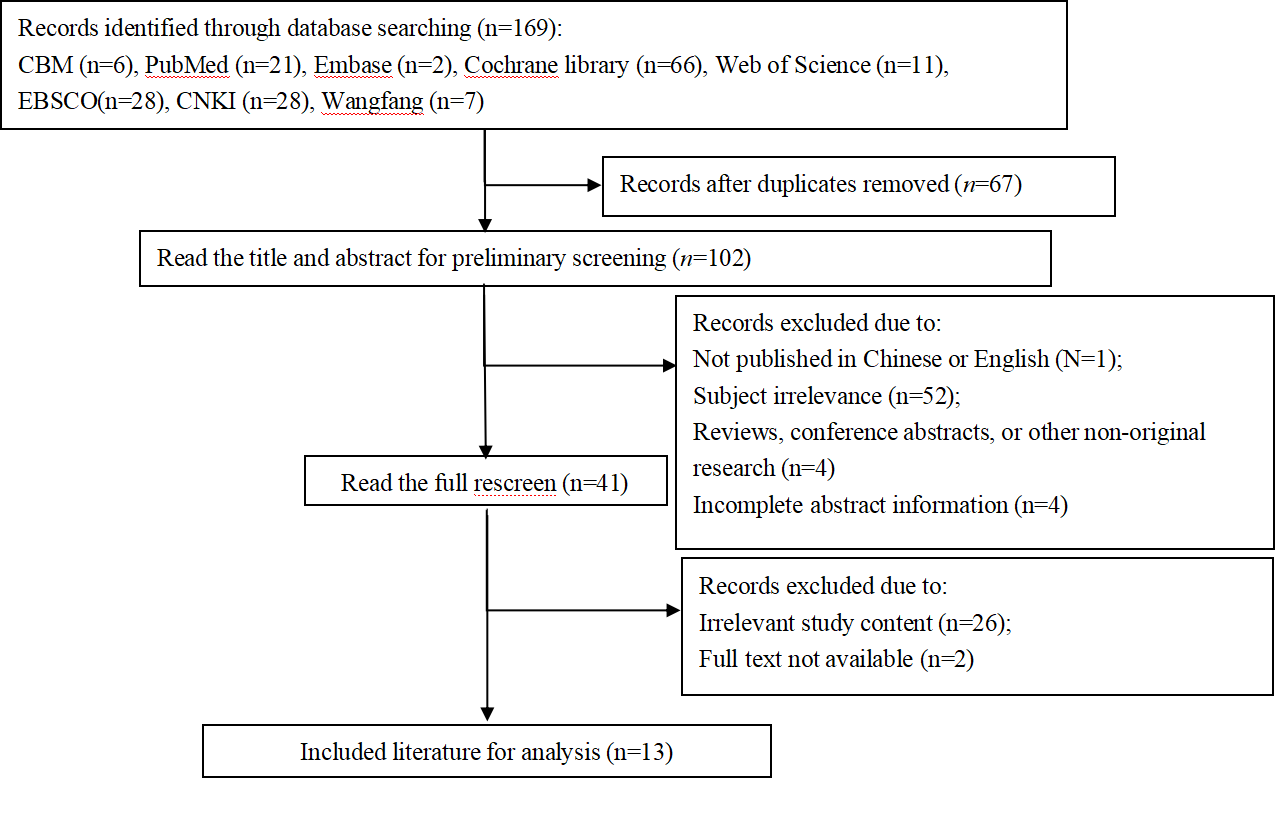

Supplement: S1 Fig — (DOCX) [file pone.0336793.s001.docx]
