# Supplementary material for: Symptom network analysis in breast cancer patients: A scoping review
Source: PLoS One. 2025 Nov 24;20(11):e0336793. doi: 10.1371/journal.pone.0336793 (PMC12643262; doi:10.1371/journal.pone.0336793)
Supplement: S1 Table — (DOCX) [file pone.0336793.s002.docx]

**S1 Table 1. Basic Characteristics of the Included Studies (n = 13).**

| Included literatures | Year | Country | Study design | Population description | Sample size | Symptom assessment tools |
| --- | --- | --- | --- | --- | --- | --- |
| Zhang et al. [12] | 2024 | China | Cross-sectional | Postoperative breast cancer patients receiving chemotherapy | 327 | MDASI, FACT-B |
| He et al. [13] | 2024 | China | Cross-sectional | Breast cancer patients receiving endocrine therapy | 330 | C-BCPT |
| Zha et al. [14] | 2024 | China | Cross-sectional | Postoperative breast cancer patients receiving radiotherapy | 168 | MSAS-Ch |
| Lei et al. [15] | 2024 | China | Cross-sectional | Home-based breast cancer patients after chemotherapy discharge | 478 | MDASI-C |
| He et al. [16] | 2024 | China | Longitudinal | Breast cancer patients receiving chemotherapy | 467 | FACT-B, Breast Cancer Chemotherapy Symptom Scale |
| Cai et al. [17] | 2023 | China | Cross-sectional | Breast cancer patients (< 60 years old) receiving chemotherapy | 1033 | PROMIS-57, PROMIS-Cognitive Function Short Form |
| Jing et al. [18] | 2023 | China | Cross-sectional | Breast cancer patients receiving endocrine therapy | 613 | FACT-ES |
| Liang et al. [19] | 2024 | China | Cross-sectional | Breast cancer patients receiving chemotherapy | 468 | MDASI |
| Chang et al. [20] | 2024 | China | Cross-sectional | Breast cancer patients receiving chemotherapy | 292 | NRS, PSQI, CFS, HADS |
| He et al. [21] | 2025 | China | Cross-sectional | Breast cancer patients receiving endocrine therapy | 406 | C-BCPT |
| Kim et al. [22] | 2024 | Korea | Cross-sectional | Breast cancer patients with treatment-induced menopausal symptoms | 250 | EORTC QLQ-C30, EORTC QLQ-BR45 |
| Xiao et al. [23] | 2025 | China | Cross-sectional | Elderly breast cancer patients (≥ 65 years old) | 481 | EORTC QLQ-C30 |
| Teng et al. [24] | 2024 | China | Cross-sectional | Breast cancer patients receiving chemotherapy | 320 | MDASI-C |

MDASI: M. D. Anderson Symptom Inventory; MDASI-C: Chinese version of the M. D. Anderson Symptom Inventory; FACT-B: Functional Assessment of Cancer Therapy–Breast; C-BCPT: Chinese version of Breast Cancer Prevention Trial Symptom Checklist; MSAS-Ch: Chinese version of Memorial Symptom Assessment Scale; PROMIS: Patient-Reported Outcomes Measurement Information System; PROMIS-CF: PROMIS Cognitive Function Short Form; FACT-ES: Functional Assessment of Cancer Therapy–Endocrine Subscale; NRS: Numeric Rating Scale; PSQI: Pittsburgh Sleep Quality Index; CFS: Cancer Fatigue Scale (Chinese version); HADS: Hospital Anxiety and Depression Scale; EORTC QLQ-C30: European Organization for Research and Treatment of Cancer Quality of Life Questionnaire–Core 30; EORTC QLQ-BR45: EORTC QLQ Breast Cancer Module.
