# Supplementary material for: Symptom network analysis in breast cancer patients: A scoping review
Source: PLoS One. 2025 Nov 24;20(11):e0336793. doi: 10.1371/journal.pone.0336793 (PMC12643262; doi:10.1371/journal.pone.0336793)
Supplement: S2 Table — (DOCX) [file pone.0336793.s003.docx]

**S2 Table 2. Core Symptoms, Bridging Symptoms and Research Methods Covered in the Literature (n = 13).**

| Included literatures | Core symptoms | Subgroup core symptoms | Research Methods for Core Symptoms | Bridge symptoms | Subgroup bridge symptoms | Research methods for bridge symptoms |
| --- | --- | --- | --- | --- | --- | --- |
| Zhang et al. [12] | — | — | — | Fatigue, poor appetite, distress | — | Coefficient of Bridges, Bridge Strength |
| He et al. [13] | Difficulty concentrating | — | Strength | Fatigue, weight gains, lack of interest in sexual activity | — | Bridge Dtrength |
| Zha et al. [14] | Fatigue | — | Strength | Skin changes | — | Betweenness |
| Lei et al. [15] | Fatigue, Sadness, Nausea | — | Closeness | fatigue, anguish, anorexia | — | Betweenness |
| He et al. [16] | Sleep difficulty (pre-chemo), Appetite loss & pain (post 1^st^ chemo), fatigue (post 3^rd^ & 6^th^ chemo) | — | Strength, Closeness, Betweenness | Depression (pre-chemo), headache (post 1^st^ chemo), fatigue (post 3^rd^ & 6^th^ chemo) | — | Betweenness |
| Cai et al. [17] | Fatigue | Helplessness and fatigue (severe); pain and despair (moderate anxiety-depression-pain); pain and fatigue (mild) | Strength, Closeness | — | — | — |
| Jing et al. [18] | Mood swings, irritability | — | Strength | — | — | — |
| Liang et al. [19] | Fatigue | — | Strength, Closeness, Betweenness | Sleep disturbance, appetite loss | — | Expected Influence, Coefficient of Bridges, Bridge Strength, Bridge Compactness |
| Chang et al. [20] | Panic | Tension and pain (high burden); Panic (low burden) | Strength, Closeness | — | — | — |
| He et al. [21] | Difficulty in concentrating | Fatigue (low distress); Pain (high distress) | Strength | — | — | — |
| Kim et al. [22] | Fatigue | — | Strength, Closeness | Fatigue | — | Betweenness |
| Xiao et al. [23] | Fatigue | — | Expected impact coefficient | — | — | — |
| Teng et al. [24] | — | Appetite loss (low self-advocacy); distress (moderate & high self-advocacy) | Strength、Closeness, Betweenness | — | — | — |
